# Serum 25-Hydroxyvitamin D and the Risk of Hip and Nonspine Fractures in Older Men

**DOI:** 10.1359/jbmr.090826

**Published:** 2009-08-31

**Authors:** Jane A Cauley, Neeta Parimi, Kristine E Ensrud, Douglas C Bauer, Peggy M Cawthon, Steven R Cummings, Andrew R Hoffman, James M Shikany, Elizabeth Barrett-Connor, Eric Orwoll

**Affiliations:** 1 University of PittsburghPittsburgh, Pennsylvania; 2 California Pacific Medical Center Research InstituteSan Francisco, California; 3 University of MinnesotaMinneapolis, Minnesota; 4 Stanford UniversityPalo Alto, California; 5 University of Alabama at BirminghamBirmingham Alabama; 6 University of California at San DiegoSan Diego, California; 7 Oregon Health & Science UniversityPortland, Oregon

**Keywords:** vitamin D, hip fracture, nonspine fracture, older men, MrOS

## Abstract

The association between vitamin D levels and incident fractures in older men is uncertain. To test the hypothesis that low serum 25-hydroxyvitamin D [(25(OH)D] levels are associated with an increased risk of fracture, we performed a case-cohort study of 436 men with incident nonspine fractures, including 81 hip fractures, and a random subcohort of 1608 men; average follow-up time 5.3 years. Serum vitamin D_2_ and vitamin D_3_ were measured on baseline sera using mass spectrometry and summed for total vitamin D. Modified Cox proportional hazards models were used to estimate the hazard ratio (HR) of fracture with 95% confidence intervals (CIs). Multivariable models included age, clinic, season, race, height, weight, and physical activity. The mean (SD) total 25(OH)D was 24.6 (7.8) ng/mL in nonspine fracture subjects, 21.5 (7.9) ng/mL in hip fracture subjects, and 25.2 (7.8) ng/mL in controls (nonspine fracture subjects versus nonpatients, *p* = .14; hip fracture subjects versus controls, *p* < .0001). 25(OH)D levels were unrelated to nonspine fractures. One SD decrease in total 25(OH)D was associated with an increased risk of hip fracture (multivariate HR = 1.60; 95% CI 1.18–2.17). Compared with men in the top quartile of total 25(OH)D (≥28), the HR of hip fracture was 2.36 (95% CI 1.08–5.15) for men in the lowest quartile (<20) (*p* = .009 for trend). Adjusting for hip bone mineral density attenuated the association by more than 50% (*p* = .065 for trend). Low serum 25(OH)D concentrations are associated with a higher risk of hip fracture in older men. Measurement of 25(OH)D may be useful in identifying men at high risk of hip fracture. © 2010 American Society for Bone and Mineral Research.

## Introduction

Vitamin D deficiency is common in older adults,(1–3) including community-dwelling seniors hospitalized for acute hip fracture.(4) An evidence-based report on vitamin D and bone health concluded that the evidence for an association between levels of serum 25-hydroxyvitamin D [25(OH)D] and fracture was inconsistent (5) and highlighted the paucity of data on circulating vitamin D and fractures in men. Since publication of this review, one prospective study reported no association between 25(OH)D and fractures in men or women,(6) while a second reported a significantly lower risk of hip fracture in a pooled sample of men and women with 25(OH)D levels greater than 24 ng/mL.(7) Low circulating 25(OH)D levels have been linked to lower bone mineral density (BMD), faster rates of bone loss, and higher bone turnover,(1) all of which could contribute to an association between 25(OH)D and fracture. Vitamin D could contribute to fractures by influencing muscle strength and balance, both of which contribute to falls and disability.(8–10) However, a meta-analysis and at least one randomized, controlled trial did not demonstrate a reduction in falls among older men taking vitamin D supplements.(9,11) Finally, low levels of 25(OH)D have been linked to frailty and poor health status, which also could contribute to an association with fractures.(10)

We conducted a case-cohort study within the Osteoporotic Fractures in Men Study (MrOS) among 436 subjects with incident fractures, including 81 hip fractures, and a randomly selected subcohort of 1608 participants. We tested the following hypotheses: (1) Low serum 25(OH)D concentrations are associated with a higher risk of fracture, and (2) this association may be mediated by poor health status, neuromuscular function, body fat, BMD, or falls.

## Materials and Methods

### Study population

From March 2000 through April 2002, 5995 community-dwelling men at six clinical centers in the United States (Birmingham, Alabama, Minneapolis, Minnesota, Palo Alto, California, Monongahela Valley near Pittsburgh, Pennsylvania, Portland, Oregon, and San Diego, California) agreed to participate in MrOS. Eligible men were at least 65 years of age without bilateral hip replacements and able to walk without the assistance of another person. Details of the MrOS design and cohort have been published.(12,13) The institutional review board at each center approved the study protocol, and written informed consent was obtained from all participants.

### Follow-up and outcome ascertainment

Men were sent questionnaires triannually to report any fractures. All fractures were verified by medical records and confirmed by blinded central adjudicators.(14) Pathologic fractures were excluded.

### Case-cohort study design

This study is a case-cohort study nested within the prospective design of MrOS. Men without sufficient serum for vitamin D assays were excluded from all analyses. Of the 5908 eligible participants, we randomly selected 1608 men to serve as the subcohort. In this subcohort, two participants were excluded: one participant with insufficient serum and another who had 25(OH)D levels more than 3 SD above the mean (75.6 ng/mL). The resulting 1606 men constituted the subcohort for this study.(15) We observed 435 incident nonspine fractures (including 81 hip fractures) in the entire cohort over the 5.3 years of follow-up. Among these subjects, 112 individuals also were sampled within the subcohort. The total study sample for the nonspine fracture analysis was 1929 men (1494 subcohort and 435 fracture cases) and for hip fracture analysis 1665 men (1584 subcohort and 81 hip fracture cases).

### Vitamin D assays

Fasting morning blood was collected, and sera were protected from sunlight and stored at −70°C until thawed for vitamin D assays. Measures of vitamin D_2_ and vitamin D_3_ were performed at the Mayo Clinic using mass spectrometry.(16) Deuterated stable isotope (d3-25-hydroxyvitamin D) was added to a 0.2 mL serum sample as internal standard. Vitamin D_2_, vitamin D_3_, and the internal standard were extracted using acetonitrile precipitation. The extracts then were further purified online and analyzed by liquid chromatography–mass spectrometry/mass spectrometry (LC-MS/MS) using multiple reaction monitoring. Vitamin D_2_ and vitamin D_3_ were quantified, reported individually, and summed for total 25(OH)D. The minimum detectable limit for vitamin D_2_ was 4 ng/mL; vitamin D_3_, 2 ng/mL. The interassay coefficient of variation (CV) was 4.4%; intraassay CV, 4.9%. Vitamin D *deficiency* was defined as total 25(OH)D < 20 ng/mL; *insufficiency*, as 21 to 29 ng/mL; *sufficiency*, as >30 ng/mL.(17)

### Measurement of BMD and body composition

Bone mineral density (BMD, g/cm^2^) of the total hip was measured using dual-energy x-ray absorptiometry (DXA; QDR 4500W, Hologic, Inc., Bedford, MA, USA). Standardized procedures for participant positioning and scan analysis were used. All DXA operators were centrally certified. Densitometry technicians at the coordinating center reviewed a random sample of all the scans, scans with exceptionally high or low BMD, and potentially problematic scans flagged at the clinic to ensure adherence to standardized techniques. Percent body fat was measured using whole-body DXA scans.

### Other measures

All covariates were assessed at baseline. Questionnaires ascertained information on date of birth, race/ethnicity, history of fracture, smoking, self-rated health, alcohol consumption, history of falls 12 months before baseline, and weight at age 25. Physical activity was assessed by asking men if they walked for exercise. Grip strength was measured twice by a handheld dynamometer (Jamar, Sammons Preston Rolyan, Bolingbrook, IL, USA).(18)

Time to complete chair stands and ability to stand from a chair without using arms (yes/no) were also recorded. To test dynamic balance, men were asked to stay within a narrow walking path (20 cm) over 6 m. Men with two or fewer deviations from the path were considered to have successfully completed the trial, and a time for completion was recorded. A participant was considered unable to complete this measure if he had no successful trials after three attempts. Height (cm) was measured on Harpenden stadiometers, and weight (kg) was measured on a standard balance beam or digital scale using standard protocols, with participants wearing light clothing without shoes. Body mass index (BMI) was calculated as kilograms per square meter (kg/m^2^).

A modified Block Food Frequency Questionnaire was administered to assess usual dietary and supplement intake over the past year (Block Dietary Data Services, Berkeley, CA, USA). Vitamin D and calcium intake were examined in these analyses. Values for participants who reported a total of less than 400 kcal/day were recorded as missing. At baseline, participants were asked to bring in all medications used within the last 30 days. All prescription medications recorded by the clinics were stored in an electronic medications inventory database. Each medication was matched to its ingredient(s) based on the Iowa Drug Information Service (IDIS) Drug Vocabulary (College of Pharmacy, University of Iowa, Iowa City, IA, USA).

### Statistical methods

Baseline characteristics were compared in the subcohort across quartiles of total 25(OH)D using chi-square tests for categorical variables and ANOVA for continuous variables. Wilcoxon nonparametric tests were used for skewed covariates. Baseline characteristics were also compared between fracture patients and the random subcohort. Baseline characteristics that were associated with fracture and 25(OH)D at *p* < .05 were identified as confounders. Other covariates known to be confounders from the literature also were selected. Associations between total serum 25(OH)D, vitamin D_3_, and vitamin D_2_ levels and incident fracture were assessed in proportional hazards regression models modified for the case-cohort design. The base model included adjustment for age, clinic, season, race, height, weight, and physical activity.

Hazard ratios (HRs) and 95% confidence intervals (CIs) were calculated per standard deviation (SD), and per 10 ng/mL decrease in total 25(OH)D and vitamin D_3_ and across quartiles defined on the basis of the distribution in the random cohort. The highest quartile formed the referent group. Vitamin D_2_ was undetectable in most men;(19) we compared fracture risk in men with detectable versus nondetectable vitamin D_2_. To assess for nonlinear trends, restricted cubic spline Cox proportional hazard models were used to examine the relationship between 25(OH)D levels and nonspine and hip fractures over the full range of 25(OH)D and vitamin D_3_.(20) Knots were chosen at the 5th, 25th, 75th, and 95th percentiles, and the reference group in these graphs was set to 95th percentile of the 25(OH)D level. Threshold effects were performed by creating a spline variable at a prespecified 25(OH)D level of 20 ng/mL, and a test of equality was done to determine if the slopes above and below the cut point were equal. All spline graphs were adjusted for the covariates in the base model.

To investigate mechanisms by which 25(OH)D might be associated with fracture, we constructed the multivariate base model and then added the following variables one at a time to determine their impact on the associations between 25(OH)D and hip fracture: (1) percent body fat, (2) health status, (3) neuromuscular function and balance (i.e., grip strength, unable to complete narrow walk or complete chair stand), (4) BMD, and (5) fall history (yes/no). We hypothesized that the association between 25(OH)D and fracture would be reduced after adjusting for these factors if they are in the causal pathway. Because no clear cut point has been published in the literature, a reduction of 10% of the HR was used as support for the hypothesis of mediation. The percentage reduction in HR was calculated as [(HR_model1_ – HR_model2_)/(HR_model1_ – 1)] × 100.

## Results

Men with the highest total 25(OH)D were younger; more likely to be Caucasian; had lower body weight, BMI, and percent body fat; were taller; had better neuromuscular performance and BMD; and also were more likely to take vitamin D and calcium supplements and had higher intakes of dietary D and calcium than men with the lowest 25(OH)D (Table 1). There were no differences in smoking, personal or family history of fracture, or falls.

**Table 1:** Baseline Characteristics of Men in the Subcohort Across Quartiles of Total 25(OH)D (ng/mL)

	Random cohort (*n* = 1606)				
		
Characteristic	Quartile 1 (3.13 to <19.0) (*n* = 394)	Quartile 2 (19.0 to <27.9) (*n* = 409)	Quartile 3 (25.1 to <27.9) (*n* = 402)	Quartile 4 (≥27.9) (*n* = 401)	*p* Value
Age (yr)	74.6 ± 6.3	73.9 ± 6.1	74.0 ± 5.7	72.7 ± 5.47	.0002
Caucasian race	324 (82.2)	370 (90.5)	371 (92.3)	376 (93.8)	<.0001
BMI (kg/m^2^)	28.1 ± 4.2	27.5 ± 3.7	27.1 ± 3.6	26.8 ± 3.1	<.0001
Weight (kg)	84.8 ± 14.5	83.6 ± 13.5	82.4 ± 12.8	82.0 ± 11.2	.0104
Weight change since age 25 (kg)	12.0 ± 12.4	10.7 ± 11.2	10.5 ± 10.9	9.2 ± 10.1	.0289
% Total body fat	27.0 ± 5.3	26.7 ± 5.3	26.1 ± 5.0	25.6 ± 5.1	.0008
Height (cm)	173.5 ± 6.9	174.3 ± 6.7	174.2 ± 7.0	175 ± 6.7	.0243
Take vitamin D supplements	29 (7.7)	52 (13.5)	46 (11.8)	72 (18.8)	.0001
Take calcium supplements	112 (29.6)	150 (38.9)	152 (39.1)	152 (39.5)	.0111
Dietary vitamin D intake (IU/day)	144.5 ± 105.9	162.8 ± 110.8	173.0 ± 122.5	166.1 ± 119.5	.0032
Dietary calcium intake (mg/day)	740.5 ± 362.0	796.1 ± 397.6	826.3 ± 391.2	812.00 ± 392.7	.0179
Diet and supplements of vitamin D (IU/day)	270.6 ± 228.4	390.9 ± 242.3	427.5 ± 246.7	454.2 ± 238.6	<.0001
Diet and supplements of calcium (mg/day)	987.9 ± 545.9	1151.2 ± 599.0	1206.3 ± 580.9	1243.9 ± 625.0	<.0001
Currently smoking	21 (5.3)	17 (4.2)	12 (3.0)	9 (2.2)	.1033
History of fracture after age 50	91 (23.1)	100 (24.5)	80 (20.0)	92 (23.0)	.3818
Parental history of fracture	115 (39.5)	140 (44.4)	136 (44.7)	141 (45.2)	.4676
History of falls in past 12 months	79 (20.1)	87 (21.3)	79 (19.7)	85 (21.2)	.9206
Self-reported health status^a^	318 (80.9)	343 (83.9)	345 (85.8)	363 (90.5)	.0014
Total hip BMD (g/cm^2^)	0.95 ± 0.15	0.94 ± 0.14	0.96 ± 0.14	0.96 ± 0.14	.0467
Oral corticosteroid use	12 (3.8)	9 (2.6)	12 (3.7)	10 (3.2)	.8334
Unable to complete chair stands	14 (3.6)	14 (3.4)	6 (1.5)	6 (1.5)	.0881
6 meter usual pace (m/s)	1.2 ± 0.3	1.3 ± 0.2	1.2 ± 0.2	1.3 ± 0.2	<.0001
Unable to attempt 20 cm narrow pace	65 (16.5)	42 (10.3)	42 (10.5)	20 (5.0)	<.0001
Maximum grip strength (kg)	40.2 ± 8.3	41.2 ± 8.0	41.5 ± 8.1	42.7 ± 8.3	.0005
Takes walks for exercise	171 (43.4)	205 (50.1)	200 (56.6)	227 (56.6)	.0031

Mean ± SD or *n* (%).

a Percent excellent/good.

Men who experienced any nonspine fracture were older, more likely to be white, shorter in stature, more likely to report a personal and parental fracture and falling in past 12 months, and had lower BMD and worse neuromuscular function than men who did not experience fracture (Table 2). Men who experienced a hip fracture also were older, had a lower BMI and body weight, had gained less weight since age 25, and had a lower vitamin D intake and BMD and worse neuromuscular function than men without a hip fracture. There were no differences in smoking, self-reported health status, use of corticosteroids, or use of calcium or vitamin D supplements or dietary calcium between fracture patients and nonpatients.

**Table 2:** Baseline Characteristics Comparing Men Who Experienced a Fracture With Men Who Were Fracture Free

Baseline characteristics	No nonspine fracture (*n* = 1494)	Nonspine fracture (*n* = 435)	No hip fracture (*n* = 1584)	Hip fracture (*n* = 81)
Age, mean (year)	73.7 ± 5.9	75.5 ± 6.5****	73.7 ± 5.9	79.8 ± 5.9****
Caucasian race	1337 (89.5)	410 (94.3)**	1419 (89.6)	77 (95.1)
BMI (kg/m^2^)	27.4 ± 3.7	27.2 ± 4.0	27.4 ± 3.7	26.5 ± 3.8*
Weight (kg)	83.4 ± 13.1	82.2 ± 13.9	83.3 ± 13.1	78.9 ± 12.6**
Weight change since age 25 (kg)	10.7 ± 11.2	10.2 ± 12.6	10.7 ± 11.1	7.0 ± 12.9*
Percent total body fat	26.3 ± 5.2	26.7 ± 5.9	26.4 ± 5.2	26.3 ± 5.8
Height (cm)	174.4 ± 6.8	173.6 ± 7.2	174.3 ± 6.9	172.4 ± 6.3*
Season of blood draw				
Winter	304 (20.4)	79 (18.2)	317 (20.0)	14 (17.3)
Spring	386 (25.8)	119 (27.4)	411 (26.0)	24 (29.6)
Summer	430 (28.8)	136 (31.3)	455 (28.7)	26 (32.1)
Fall	374 (25.0)	101 (23.2)	401 (25.3)	17 (21.0)
Take vitamin D supplements	186 (13.0)	64 (15.7)	195 (12.9)	11 (14.7)
Take calcium supplements	527 (36.8)	167 (40.4)	559 (36.8)	29 (38.7)
Dietary vitamin D intake (IU/day)	162.9 ± 116.2	160.1 ± 106.7	162.0 ± 115.2	144.3 ± 108.2
Dietary calcium intake (mg/day)	796.8 ± 390.3	773.4 ± 374.2	794.1 ± 387.3	744.1 ± 426.6
Diet and supplements of vitamin D (IU/day)	390.3 ± 249.3	376.0 ± 240.3	387.5 ± 248.6	323.4 ± 237.9*
Diet and supplements of calcium (mg/day)	1151.1 ± 600.0	1152.4 ± 595.6	1148.1 ± 595.9	1118.3 ± 681.8
Currently smoking	55 (3.7)	15 (3.5)	58 (3.7)	4 (4.9)
History of fracture after age 50	319 (21.4)	175 (40.2)****	352 (22.3)	39 (48.2)****
Parental history of fracture	488 (42.9)	167 (50.6)*	522 (43.3)	30 (50.0)
History of falls within the past 12 months	289 (19.3)	142 (32.6)****	323 (20.4)	25 (30.9)*
Good to excellent health status	1272 (85.2)	365 (83.9)	1352 (85.4)	65 (80.3)
Total hip BMD (g/cm^2^)	0.96 ± 0.14	0.89 ± 0.15****	0.96 ± 0.14	0.79 ± 0.14****
Oral corticosteroid use	41 (3.4)	10 (2.8)	42 (3.3)	2 (3.0)
Unable to complete chair stands	38 (2.6)	25 (5.8)***	38 (2.4)	11 (13.8)****
Unable to attempt 20 cm narrow pace	146 (9.8)	70 (16.1)***	161 (10.2)	20 (24.7)****
Maximum grip strength (kg)	41.6 ± 8.2	39.7 ± 9.0***	41.5 ± 8.2	36.6 ± 7.8****
Takes walks for exercise	747 (50.0)	197 (45.3)	793 (50.1)	34 (42.0)

Mean ± SD or *n* (%).

* *p* < .05 vs. controls.

** *p* < .01 vs. controls.

*** *p* < .001 vs. controls.

**** *p* < .0001 vs. controls.

### Serum vitamin D and fracture

The mean total 25(OH)D level was 16% lower in hip fracture patients than in the subcohort, but there was no difference in total 25(OH)D levels between men with any nonspine fracture versus controls (Table 3). Almost half the men who suffered a hip fracture had total 25(OH)D levels in the deficient range (<20.0 ng/mL; 7% had values less than 10 ng/mL). Vitamin D_3_ levels were 15% lower among men who suffered a hip fracture compared with controls. A slightly lower proportion of hip fracture patients did not have measurable vitamin D_2_ levels, but this was not statistically significant.

**Table 3:** Serum 25(OH)D Levels (ng/mL) by Fracture Status

	No nonspine fracture	Nonspine fracture	No hip fracture	Hip fracture
Total 25(OH)D				
Mean ± SD	25.16 (7.85)	24.55 (7.76)	25.15 (7.89)	21.15 (7.92)**
Median (range)	25.10 (3.13–58.30)	24.80 (4.36–55.2)	25.1 (3.10–58.3)	20.6 (4.4–40.6)
<20 ng/mL, *n* (%)	372 (24.90)	116 (26.67)	395 (24.94)	37 (45.68)**
20 to <30 ng/mL, *n* (%)	755 (50.54)	225 (51.72)	797 (50.32)	33 (40.74)
≥30 ng/mL, *n* (%)	367 (24.56)	94 (21.61)	392 (24.75)	11 (13.58)
Vitamin D_3_				
Mean ± SD	22.91 (8.31)	22.37 (8.27)	22.95 (8.34)	19.61 (8.08)*
Median (range)	22.40 (2.40–58.30)	22.40 (4.20–55.20)	22.6 (2.4–58.3)	19.2 (4.4–40.6)
Vitamin D_2_				
>0 ng/mL, *n* (%)	396 (26.51)	113 (25.98)	417 (26.33)	14 (17.28)

* *p* < .001 versus controls.

** *p* = .0001 versus controls.

Total 25(OH)D, vitamin D_3_, and vitamin D_2_ were unrelated to the risk of nonspine fracture (Table 4). The adjusted HR for incident nonspine fracture per 1 SD decrease in total 25(OH)D was 1.07 (95% CI 0.96–1.21). To further explore the relationship of vitamin D to nonspine fracture, we stratified by age. There was no evidence of an association between vitamin D and nonspine fracture even in the oldest (age ≥ 80 years; data not shown).

**Table 4:** Relative Hazard (RH) (95% CI) of Nonspine Fracture or Hip Fracture by Serum 25(OH)D Levels (ng/mL)

	Nonspine fracture RH (95% CI)^a^	Hip fracture RH (95% CI)^a^
Total 25(OH)D		
Per SD (7.9)	1.07 (0.96–1.21)	1.60 (1.18–2.17)
Per 10 ng/mL	1.11 (0.94–1.27)	1.85 (1.27–2.69)
Quartiles		
1st (3.13 to <19.0)	1.21 (0.86–1.65)	2.36 (1.08–5.16)
2nd (19.0 to <25.1)	1.15 (0.84–1.56)	1.48 (0.68–3.21)
3rd (25.1 to <27.9)	1.13 (0.81–1.53)	0.98 (0.42–2.28)
4th (≥27.9)	Referent	Referent
*p* trend	.2927	.0093
Vitamin D_3_		
Per SD (8.3)	1.06 (0.95–1.19)	1.42 (1.05–1.91)
Per 10 ng/mL	1.08 (0.94–1.23)	1.52 (1.06–2.16)
Quartiles		
1st (2.07 to <17.0)	1.17 (0.86–1.61)	1.54 (0.77–3.09)
2nd (17.0 to <22.4)	1.02 (0.74–1.40)	1.41 (0.71–2.80)
3rd (22.4 to <28.2)	1.19 (0.88–1.62)	0.57 (0.25–1.34)
4th (≥28.2)	Referent	Referent
*p* trend	.55	.049
Vitamin D_2_	1.00	1.00
>0 vs. 0	1.01 (0.78–1.28)	0.62 (0.33–1.17)
*p* value	0.97	0.14

a Base model adjusting for age, race, clinic, season of blood draw, physical activity, height, and weight.

In contrast, low total 25(OH)D levels were associated with a higher risk of hip fracture; the adjusted HR of hip fracture per 1 SD decrease in total 25(OH)D was 1.60 (95 % CI 1.18–2.17). Men with the lowest total 25(OH)D levels had a greater than twofold increased risk of hip fracture compared with men with the highest total 25(OH)D levels (HR = 2.36; 95% CI 1.08–5.16; *p* = .009 for trend).

The association between vitamin D_3_ and fractures was similar but weaker. A 1 SD decrease in vitamin D_3_ was associated with a 42% increased risk of hip fracture. Men in the lowest quartile of vitamin D_3_ had a 54% increased risk of hip fracture compared with men with the highest vitamin D_3_ level (*p* = .049 for linear trend). Men with measurable vitamin D_2_ had a lower risk of hip fracture, but confidence intervals were wide, and this association was not significant.

**Fig. 1:**
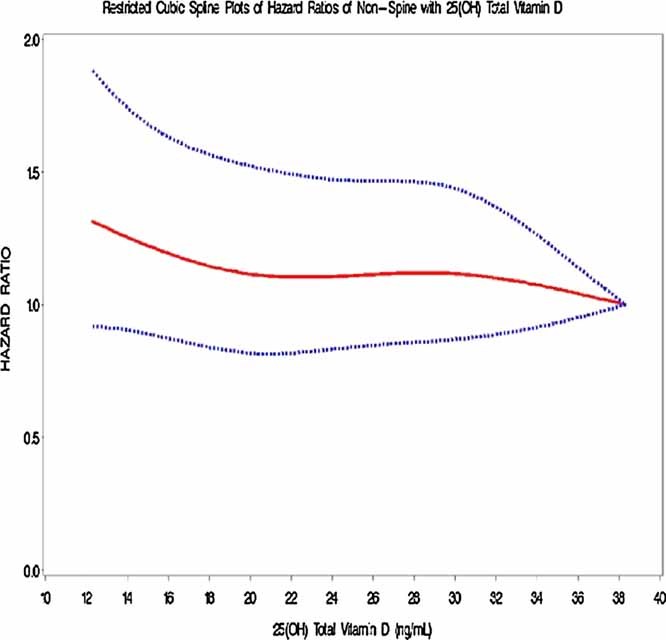
Restricted cubic spline plots of hazard ratios of nonspine with 25(OH)D.

**Fig. 2:**
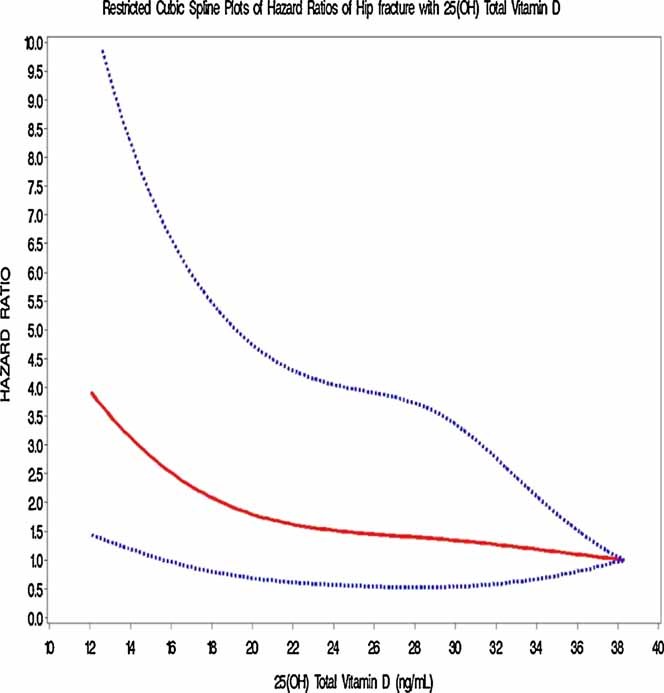
Restricted cubic spline plots of hazard ratios of hip fracture with 25(OH)D.

Examination of the association over the full range of total 25(OH)D levels revealed no association with nonspine fracture (Fig. 1). For hip fractures, there was no evidence of a threshold (for test for nonequal slopes, *p* = .59), although the slope for hip fractures was steepest in men with a total 25(OH)D less than 20 ng/mL (Fig. 2).

### Potential mediators

Inclusion of percent body fat, health status, or neuromuscular function in the models reduced the HR by 22%, 10%, and 18%, respectively, suggesting some mediating role of these parameters in the relationship between total 25(OH)D and hip fracture (Table 5). However, total hip BMD reduced the HR by more than 50%, suggesting a major role for BMD in mediating this association. Inclusion of baseline information on falls had no effect on the HR.

**Table 5:** Relative Hazard (95% CI) of Hip Fracture According to Total 25(OH)D and Vitamin D_3_ Levels: Effect of Adjustment for Possible Mediators

	Quartile of Total 25(OH)D (ng/mL)				
		
	1st (3.13 to <19.0)	2nd (19.0 to <25.1)	3rd (25.1 to <29.9)	4th (≥29.9)	*p* for linear trend
Total 25(OH)D					
Base^a^	2.36 (1.08–5.15)	1.48 (0.68–3.21)	0.98 (0.42–2.28)	Ref	.009
Base^a^ + percent of body fat	2.06 (0.93–4.57)	1.40 (0.64–3.08)	0.91 (0.39–2.14)	Ref	.029
Base^a^ + health status	2.22 (1.02–4.83)	1.40 (0.64–3.06)	0.95 (0.41–2.22)	Ref	.014
Base^a^ + neuromuscular measures^b^	2.11 (0.94–4.75)	1.45 (0.66–3.22)	0.86 (0.35–2.12)	Ref	.019
Base^a^ + hip BMD	1.64 (0.73–3.68)	0.99 (0.44–2.23)	0.68 (0.27–1.68)	Ref	.065
Base^a^ + falls	2.38 (1.09–5.20)	1.51 (0.69–3.26)	0.99 (0.42–2.30)	Ref	.009
Vitamin D_3_					
Base	1.54 (0.77–3.09)	1.41 (0.71–2.91)	0.57 (0.25–1.34)	Ref	.049
Base^a^ + percent of body fat	1.35 (0.66–2.77)	1.44 (0.71–2.92)	0.61 (0.26–1.44)	Ref	.128
Base^a^ + health status	1.50 (0.75–3.00)	1.32 (0.66–2.64)	0.56 (0.24–1.32)	Ref	.057
Base^a^ + neuromuscular measures^b^	1.44 (0.68–3.05)	1.37 (0.67–2.81)	0.59 (0.24–1.45)	Ref	.103
Base^a^ + Hip BMD	1.03 (0.48–2.22)	0.98 (0.49–1.97)	0.46 (0.20–1.08)	Ref	.387
Base^a^ + falls	1.56 (0.78–3.14)	1.44 (0.72–2.86)	0.57 (0.24–1.34)	Ref	.045

a Base model adjusting for age, race, clinic, season of blood draw, physical activity, weight, and height.

b Neuromuscular measures: unable to complete chair stands or narrow walk; grip strength.

For vitamin D_3_, inclusion of percent body fat and neuromuscular measures reduced the HR by 35% and 17%, respectively. Inclusion of health status or falls in the models reduced the HR by less than 10%. Adjustment for total hip BMD completely attenuated the association between and vitamin D_3_ and hip fracture.

## Discussion

In our prospective case-cohort study, we found that men with the lowest total 25(OH)D concentration (<19.9 ng/mL) at study entry had a significantly increased risk for subsequent hip fractures during the next 5 years over men with the highest concentrations (≥27.9 ng/mL). A similar association was observed between vitamin D_3_ and hip fracture, but vitamin D_2_ was unrelated to hip fractures. Vitamin D concentrations were not related to risk of any nonspine fractures, suggesting an association with more frailty-related fractures.

To our knowledge, few studies have examined the relationship between total 25(OH)D and fractures in older men. Five of 12 case-control studies included men, and all reported lower 25(OH)D levels in men with acute hip fracture compared with controls.(5) Only three prospective studies included men. Woo (21) studied 427 patients, including 171 men. Subjects who experienced an incident fracture had lower total 25(OH)D levels, but the relationship was not significant. The association between total 25(OH)D and fracture was studied in 730 incident fracture patients (155 men) and 1445 matched controls (309 men).(6) There was no evidence of an association between 25(OH)D and fracture in men or women. These results are consistent with our findings that 25(OH)D concentrations were unrelated to all nonspine fractures. There were too few hip fractures (*n* = 5) to examine hip fractures separately.

Our hip fracture results are consistent with a report from the Third National Health and Nutrition Examination Survey (NHANES III), in which the relative risk for hip fracture was significantly reduced in subjects with 25(OH)D concentrations greater than 25 ng/mL.(7) This study included 62 men with hip fractures and 869 control men, but results were not stratified by gender. Our results also are consistent with a recent report in women that low total serum 25(OH)D levels are associated with an increased risk of hip fracture.(22)

We studied several possible mechanisms whereby 25(OH)D concentrations may influence hip fracture risk. The increased fracture risk could be related to impaired muscle strength and balance and poor health status, all of which could increase the risk of falls and subsequently fracture.(8–10) Inclusion of health status or neuromuscular function reduced the HR by 10% and 18%, respectively, meeting our criteria for mediation. Lower bioavailability of vitamin D in obese patients and inverse associations between 25(OH)D and adiposity have been reported.(23–25) Low fat mass and body weight have been linked to an increased risk of hip fracture.(26) Hence the higher degree of adiposity in men with the lowest 25(OH)D levels could confound our results. Adjusting for percent body fat in our analyses resulted in a 22% attenuation in the HR association. Surprisingly, addition of falls had little effect on the relationship between 25(OH)D and hip fracture, perhaps because we assessed falls at baseline and did not adjust for falls over the follow-up period.

Low circulating vitamin D has been shown to affect bone strength.(1) Vitamin D deficiency in milder forms can lead to secondary hyperparathyroidism and subsequently faster rates of bone turnover and bone loss. Positive relationships have been observed between serum 25(OH)D and BMD.(27,28) Adjustment for BMD attenuated the association between total 25(OH)D and hip fracture such that the overall trend was borderline significant. Adjustment for BMD completely attenuated the relationship bewteen vitamin D_3_ level and hip fracture. This suggests that the association between low vitamin D level and increased hip fracture risk is at least in part due to lower hip BMD among the men with low vitamin D levels. This result differs from the NHANES III study, where the relationship between 25(OH)D and hip fracture was independent of BMD.(7) Low BMD is associated with an increased risk of all nonspine fractures,(29) and if BMD is the mediator, it is somewhat surprising that there was no relationship between 25(OH)D and nonspine fracture. The relationship between BMD and nonspine fracture is, however, weaker than for hip fracture. Further studies are needed to confirm the mechanism underlying the association of 25(OH)D and hip fracture. In particular, future studies should include markers of bone turnover. In women, we observed that high bone resorption may be an important mechanism for the association between low 25(OH)D and hip fracture.(22)

The optimal serum 25(OH)D concentration needed to maintain bone health has not been established. Optimal concentrations have been defined as those at which serum parathyroid hormone levels plateau in the normal range;(30) however, this definition has led to a wide range of optimal 25(OH)D concentration thresholds (8–46 ng/mL). More recently, the optimal threshold concentration of 25(OH)D, based on BMD levels, was found to be at least 31 ng/mL, with a target of 37–42 ng/mL.(28) Randomized trials of vitamin D supplementation that brought mean serum 25(OH)D concentrations up to 30–41 ng/mL found significantly lower fracture rates.(31) Trials in which the mean serum 25(OH)D concentration did not reach this threshold showed no effect on fractures.(32,33) Our results showed the greatest risk of hip fracture among men with a 25(OH)D level of less than 19.9 ng/mL, consistent with our recent findings among older women.(22) This suggests a somewhat lower threshold for defining “optimal” levels by hip fracture risk, although the threshold model was not significant, and we had limited power to detect a threshold.

Our study is unique in the measurement of total 25(OH)D, vitamin D_2_, and vitamin D_3_. Results generally were similar between total 25(OH)D and vitamin D_3_, albeit a bit weaker for vitamin D_3_. We found no relationship with vitamin D_2_, but the majority of men did not have measurable vitamin D_2_. The major determinant of having measurable vitamin D_2_ was self-report of vitamin D supplements.(19) At the time that the serum was collected, vitamin D supplementation was limited primarily to vitamin D_2_. This implies that the level of supplement used by these men was ineffective in preventing hip fractures. Nevertheless, the clinical advantage of measuring both vitamin D_2_ and vitamin D_3_ separately is uncertain and has been shown to cause some clinical confusion.(34) Results were strongest for total 25(OH)D, and it is currently recommended that separate reporting should not be carried out until the clinical utility has been demonstrated.

Our study has several limitations. Nonwhite men are more likely to be vitamin D deficient;(17) however, most of the fractures occurred in white men. We do not have measures of parathyroid hormone, which could contribute to the relationship between 25(OH)D and hip fracture.(1) Finally, few men had 25(OH)D concentrations greater than 30 ng/mL, so we could not test whether even higher concentrations offer greater protection against hip fracture.

Despite these limitations, we conclude that low serum 25(OH)D concentrations are associated with an increased risk of hip fracture in community-dwelling men. Low BMD may contribute to this association. Similar to our results in older women,(22) our findings suggest that low serum 25(OH)D concentrations may help to identify men at high risk of hip fracture.
